# The transcriptome landscape of early maize meiosis

**DOI:** 10.1186/1471-2229-14-118

**Published:** 2014-05-03

**Authors:** Stefanie Dukowic-Schulze, Anitha Sundararajan, Joann Mudge, Thiruvarangan Ramaraj, Andrew D Farmer, Minghui Wang, Qi Sun, Jaroslaw Pillardy, Shahryar Kianian, Ernest F Retzel, Wojciech P Pawlowski, Changbin Chen

**Affiliations:** 1 Department of Horticultural Science, University of Minnesota, St. Paul, MN 55108, USA; 2 National Center for Genome Resources, Santa Fe, NM 87505, USA; 3 Department of Plant Breeding and Genetics, Cornell University, Ithaca, NY 14850, USA; 4 Computational Biology Service Unit, Cornell University, Ithaca, NY 14850, USA; 5 USDA-ARS Cereal Disease Laboratory, University of Minnesota, St. Paul, MN 55108, USA

**Keywords:** Maize, Meiosis, Meiocytes, Mitochondria, RNA-seq, Transcriptome

## Abstract

**Background:**

A major step in the higher plant life cycle is the decision to leave the mitotic cell cycle and begin the progression through the meiotic cell cycle that leads to the formation of gametes. The molecular mechanisms that regulate this transition and early meiosis remain largely unknown. To gain insight into gene expression features during the initiation of meiotic recombination, we profiled early prophase I meiocytes from maize *(Zea mays)* using capillary collection to isolate meiocytes, followed by RNA-seq.

**Results:**

We detected ~2,000 genes as preferentially expressed during early meiotic prophase, most of them uncharacterized. Functional analysis uncovered the importance of several cellular processes in early meiosis. Processes significantly enriched in isolated meiocytes included proteolysis, protein targeting, chromatin modification and the regulation of redox homeostasis. The most significantly up-regulated processes in meiocytes were processes involved in carbohydrate metabolism. Consistent with this, many mitochondrial genes were up-regulated in meiocytes, including nuclear- and mitochondrial-encoded genes. The data were validated with real-time PCR and *in situ* hybridization and also used to generate a candidate maize homologue list of known meiotic genes from Arabidopsis.

**Conclusions:**

Taken together, we present a high-resolution analysis of the transcriptome landscape in early meiosis of an important crop plant, providing support for choosing genes for detailed characterization of recombination initiation and regulation of early meiosis. Our data also reveal an important connection between meiotic processes and altered/increased energy production.

## Background

Meiosis is a key process in the life cycle of higher plants during which recombination occurs, leading to novel combinations of parental alleles. Many of the meiotic genes that are well characterized to date are directly involved in the meiotic recombination machinery and identifying the entire set of meiotic genes is an on-going process. In the well-studied model dicot plant, *Arabidopsis thaliana,* around 70 genes involved in meiosis have been functionally characterized [1-6]. In crop plants there are few well-characterized meiotic genes, but attempts have been made in maize, rice, wheat and barley to generate a comprehensive atlas of meiotic genes corresponding to well-characterized homologs from other organisms [7,8]. Several transcriptome studies using whole anthers have been performed in species such as Arabidopsis [9], petunia [10], rice [11-13], hexaploid wheat [14] and maize [15,16]. Studies on multiple stages during anther development have yielded valuable data on transcriptome dynamics and stage-specific transcripts [10,13-15]. In addition, some studies have helped to elucidate the meiotic transcriptome by comparing meiotic mutant anthers to wild-type [9,16-19].

However, these studies examined transcriptomes of whole anthers, which, while technically much less challenging than isolating meiocytes (cells undergoing meiosis), does not distinguish between meiocyte gene expression and gene expression in the various other tissues of the anther. Comparison with meiotic mutant anthers improves this but can suffer from distortion in tissue composition and in gene expression caused by the mutation. To gain insight specifically into the transcriptome of meiocytes, recent efforts involved techniques for isolation of pure meiocytes. Obtaining early meiocytes from plants is possible by using CCM (**C**apillary **C**ollection of **M**eiocytes), in which meiocytes are collected with a microcapillary [20]. In these studies, mRNA of isolated Arabidopsis meiocytes at stages ranging from prophase I to tetrads was analyzed using expression microarrays [21] or next-generation sequencing [5,22]. These studies found that a large number of Arabidopsis genes were expressed during meiosis [5,22]. Interestingly, meiocytes also showed substantial expression of transposable elements [5,22] as well as high levels of transcripts mapping to a mitochondrial genome insertion (MGI) in the nuclear genome [5]. While these studies were based on pooled cells from all meiotic stages, no study has previously examined specifically the early meiotic transcriptome within isolated meiocytes. Early steps of meiosis during meiotic prophase I are the stages when spore mother cells have left the mitotic cell cycle and entered the meiotic cell cycle, and chromosomes start to pair and recombine [1,6,23]. These processes are critical for the success of meiosis. Identifying genes and processes that are specifically enriched is important for understanding the molecular mechanisms of regulation in early meiosis, recombination initiation, and gamete formation.

In this study, we took advantage of the synchrony of development that exists in the male inflorescence (tassel) in maize (*Zea mays*) to collect large quantities of meiocytes at leptotene and zygotene sub-stages of prophase I. We took a closer look at the early meiotic transcriptome of isolated meiocytes with two main objectives: First, we wanted to complement previous studies with a list of meiotic gene candidates in maize, reporting their expression level in both isolated meiocytes and anthers. Our second goal was to take a more general approach and reveal important processes during early meiosis beyond those directly involved in the conserved process of recombination.

## Results

### Gene expression profile of isolated male maize meiocytes

**Figure 1:**
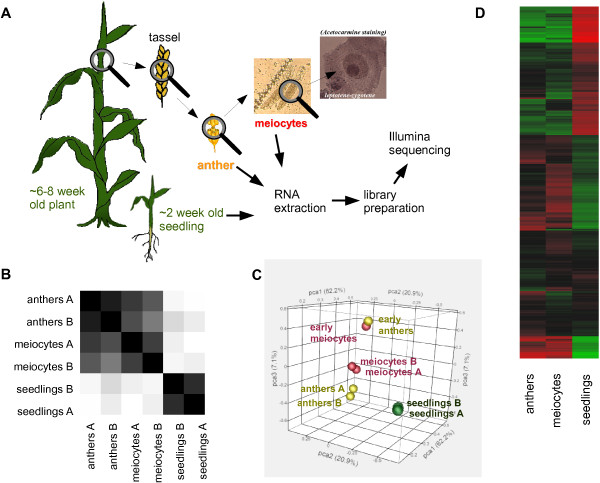
**Correlation between RNA-seq samples of *Zea mays *B73. (A)** Experimental approach. **(B)** Dendrogram of hierarchical clustering analysis for correlation between the samples. **(C)** Principal Component Analysis (PCA) Plot for pattern discovery. Normalized, trimmed data of the replicates, compared with additional samples of early meiocytes and early anthers. **(D)** Heatmap of gene expression levels. Log_2_ values are coded on the green-to-red scale. Red = high expression level, green = low expression level.

We used CCM (**C**apillary **C**ollection of **M**eiocytes) followed by RNA extraction and Illumina sequencing to generate transcriptome profiles of isolated meiocytes at the leptotene and zygotene stages of prophase I, whole anthers containing meiocytes at the same stages, and two-week old seedlings of maize (Figure 1A). Each transcriptome was generated in two biological replicates, correlation coefficients between the replicates being 0.9174 (meiocytes), 0.9419 (anthers), and 0.7990 (seedlings; Additional file 1: Table S1). RNA yield ranged from 2.3-6.7 μg, and total sequenced reads from 36,126,210-77,551,649, with at least 18,535,914 reads aligning uniquely (Additional file 1: Table S1). A correlation dendrogram, generated by hierarchical clustering using the Ward method in JMP Genomics shows that paired biological replicates correlate best with each other (Figure 1B). There is also a high correlation between anthers and meiocytes (Figure 1B-D, Additional file 1: Table S1) and the correlation of our biological replicates is especially obvious when comparing the early prophase meiocyte and anther samples with additional premeiotic meiocyte and anther samples (Figure 1C).

**Figure 2:**
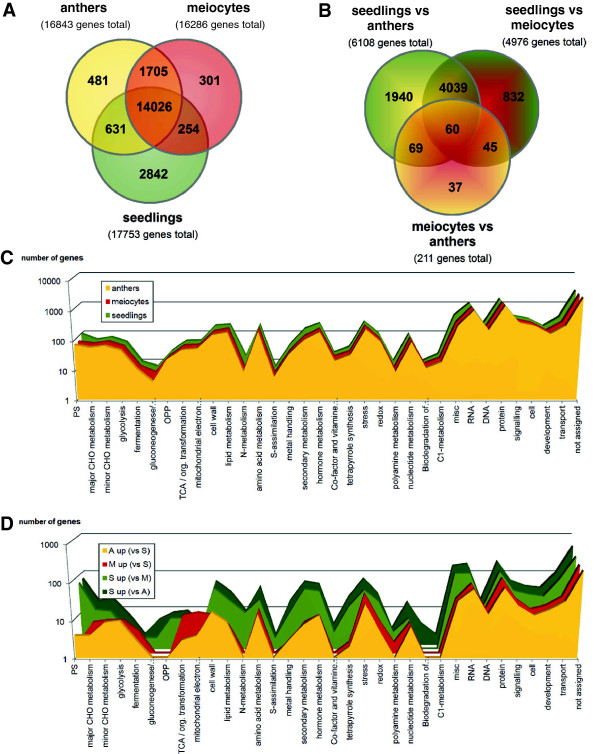
**Gene expression profiles of meiocytes, anthers and seedlings of the maize B73 inbred. (A)** Venn Diagram of all genes with at least 5 RPM per sample. **(B)** Venn Diagram of all genes differentially expressed between samples (*P*_adj_ ≤ 0.01). **(C)** GO distribution of genes shown in **(A)**. **(D)** GO distribution of differentially expressed genes shown in **(B)**. PS = photosynthesis, CHO = carbohydrate, OPP = oxidative pentose phosphate pathway, TCA = tricarboxylic acid cycle, misc = miscellaneous

Above a threshold of 5 reads per million mapped reads, 16,286 genes were expressed in meiocytes, 16,843 in anthers, and 17,753 in seedlings, with ~79-86% of them common to all samples (Figure 2A); for numbers of genes in case of 2 or 10 RPM and a comparison with equivalent *Arabidopsis* data see [24]. Few genes were uniquely expressed in one sample, namely 2% of meiocyte genes, 3% of anther genes, and 16% of seedling genes (Figure 2A). Note that anther samples contain meiocytes, which likely contributes to the small number of differentially expressed genes (both up- and down-regulated) between anthers and meiocytes; substantially more differentially expressed genes were found between anthers or meiocytes vs. seedlings (Figure 2B, Additional file 2: Figure S1A-C; lists of up- and down-regulated genes generated with the DEseq package for R Statistical Analysis, using a threshold significance of *P*_adj_ ≤ 0.01, Additional file 3: Table S2). High congruence of anthers and meiocytes in the expression heatmap also clearly sets them apart from seedlings (Figure 1D, Additional file 2: Figure S1D).

Since nearly 85% of the maize genome consists of transposable elements (TEs) [25], we analyzed differential TE expression in meiocytes, anthers and seedlings. In contrast to their relative genomic abundance, annotated TEs contribute only ~12% to global expression, while annotated genes contribute ~80%, averaged across all data sets; reads originating from regions of the genome not present in the reference or reads whose quality is too poor to allow alignment make up the remaining portion (Additional file 4: Figure S2A). We conducted a thorough analysis for TE expression in meiocytes (Additional file 4: Figure S2B-E) and detected a preference for LTRs of the [RLX] Unknown TE superfamily and a strong bias in chromosome origin of TEs expressed at higher levels during meiosis (Additional file 4: Figure S2D + E): Most meiosis-specific TEs originated from chromosome 6, and substantially more TEs originating from the mitochondrial genome were detected than in non-meiosis-specific TEs (Additional file 4: Figure S2E). The over-representation of the [RLX] Unknown TE superfamily as well as of chromosome 6 derived TEs is due to “Ipiki” family TEs (Database ID AC212468_11834), of which many are up-regulated, some up to ~200-fold in meiosis-specific TEs. 231 out of 255 Ipiki elements are located on chromosome 6, on the distal part of the short arm, distal to the nucleolus organizer region. In addition, we noticed an increased occurrence of TE families previously reported as highly expressed in meiotic or mitotic tissues by Vicient et al. [26] in our meiosis-specific TEs, especially Giepum, Cinful and Flip (Additional file 4: Figure S2F).

Subjecting all genes expressed per sample to functional annotation using MapMan [27] shows that difference between functional category distributions among the samples is minimal (Figure 2C). The only difference is seen in genes related to photosynthesis and secondary metabolism, which are enriched in seedlings (Figure 2C). No differences are apparent between anthers and meiocytes in this approach.

Subjecting genes up-regulated in our samples to MapMan for an overview of functional terms (Figure 2D), on the other hand, showed obvious differences. Most functional categories enriched in anthers are also enriched in meiocytes alone, in accordance with their similar expression profiles (Additional file 2: Figure S1D). Genes up-regulated in whole anthers vs. seedlings show enrichment for genes involved in chromatin packaging and organization, transcription, RNA biosynthetic processes, as well as regulatory processes (Figure 2D). Furthermore, common to the transcriptomes of anthers and meiocytes is a high prevalence of genes implicated in energy production, such as glycolysis, fermentation, TCA (tricarboxylic acid cycle), and mitochondrial electron transport (Figure 2D). Genes up-regulated in seedlings are enriched for those involved in photosynthesis, OPP (oxidative pentose phosphate pathway), cell wall and lipid metabolism, secondary metabolism, and nitrogen and sulfur metabolism (Figure 2D).

### Detailed GO analysis of genes up-regulated in meiocytes

To gain deeper insight into processes during early meiosis, we extended our functional analysis for genes up-regulated in meiocytes using AgriGO ([28], http://bioinfo.cau.edu.cn/agriGO/). Analysis of up- or down-regulated genes did not yield significant GO (gene ontology) terms for comparisons between meiocytes and anthers. All other comparisons returned multiple significant GO terms (Additional file 5: Table S3).

Genes up-regulated in meiocytes vs. seedlings are enriched for a few significant GO terms (Table 1, Additional file 5: Table S3) including energy- and mitochondria-related processes and various regulatory mechanisms, such as redox homeostasis and chromatin modification. The most significantly enriched GO term in the meiocytes vs. seedlings comparison is “cellular carbohydrate metabolic process”. Other GO categories enriched in meiocytes vs. seedlings are “localization” (containing genes encoding transmembrane proteins and receptors in mitochondria or ER, and RasGTPases, see Additional file 6: Figure S3A), “signaling” (including RasGTPase genes), “DNA repair” (especially genes encoding mismatch or excision repair proteins), “proteolysis” (with genes for proteasome subunits and cell-cycle-progression protein SKP1), and “glycosylation” (comprised of genes for ribophorins and galectins). Besides these highly significantly enriched GO categories, “chromatin” (with a majority of genes for histones and histone modifiers), “RNA” (with genes for transcription factors, see Additional file 6: Figure S3B, ribosomal proteins and histones) and “homeostasis” (comprised mostly of genes for thioredoxins and glutaredoxins) are also significantly enriched in meiocytes vs. seedlings but to a lower extent.

**Table 1:** Significant GO terms in genes up-regulated in meiocytes vs. seedlings

**Group**	**GO**	**Description**	**p-value**
Carbohydrate metabolism	GO:0044262*	Cellular carbohydrate metabolic process	1.00E-06
	GO:0044265*	Cellular macromolecule catabolic process	0.0002
	GO:0044248*	Cellular catabolic process	0.00024
	GO:0006066*	Alcohol metabolic process	0.0011
	GO:0019318*	Hexose metabolic process	0.0021
	GO:0005975*	Carbohydrate metabolic process	0.0061
	GO:0005996*	Monosaccharide metabolic process	0.0097
	GO:0006006*	Glucose metabolic process	0.018
	GO:0006096*	Glycolysis	0.021
Localization	GO:0033036*	Macromolecule localization	0.00016
	GO:0008104*	Protein localization	0.0021
	GO:0006605*	Protein targeting	0.01
	GO:0045184*	Establishment of protein localization	0.014
	GO:0015031*	Protein transport	0.014
DNA repair	GO:0006298*	Mismatch repair	0.00022
	GO:0006281	DNA repair	0.013
	GO:0033554	Cellular response to stress	0.014
	GO:0051716	Cellular response to stimulus	0.015
	GO:0006974	Response to DNA damage stimulus	0.015
Proteolysis	GO:0051603*	Proteolysis involved in cellular protein catabolic process	0.00034
	GO:0006511*	Ubiquitin-dependent protein catabolic process	0.00034
	GO:0044257*	Cellular protein catabolic process	0.00034
	GO:0043632*	Modification-dependent macromolecule catabolic process	0.00034
	GO:0019941*	Modification-dependent protein catabolic process	0.00034
Glycosylation	GO:0043413*	Macromolecule glycosylation	0.00055
	GO:0009100*	Glycoprotein metabolic process	0.00055
	GO:0009101*	Glycoprotein biosynthetic process	0.00055
	GO:0006486*	Protein amino acid glycosylation	0.00055
	GO:0070085*	Glycosylation	0.00055
Anion transport	GO:0006820*	Anion transport	0.011
Chromatin	GO:0051276	Chromosome organization	0.014
	GO:0016568*	Chromatin modification	0.016
RNA	GO:0009059	Macromolecule biosynthetic process	0.019
	GO:0006351	Transcription, DNA-dependent	0.022
	GO:0032774	RNA biosynthetic process	0.023
	GO:0034645	Cellular macromolecule biosynthetic process	0.027
Homeostasis	GO:0019725	Cellular homeostasis	0.02
Signaling	GO:0007264	Small GTPase mediated signal transduction	0.021

*Terms also enriched in list of genes designated as meiocyte genes.

We designated a group of 2,223 genes as meiocyte genes using the following criteria defined previously by Chen et al. [5]: gene expression level in meiocytes at least 2-fold higher than in anthers (M/A ≥ 2), or, for genes expressed at least two-fold higher in meiocytes and anthers compared to seedlings, less than 4-fold in anthers vs. meiocytes (A/M < 4, if M/S and A/S both ≥ 2). Overall, 457 genes met the first criterion, and 2,187 met the second criterion. Most (429) genes of the first group were also present in the second group, yielding 2,223 genes in the combined list. Subjecting these genes to GO analysis with AgriGO [28] identified more enrichment for terms related to “nucleosome assembly” and “DNA packaging” and several additional enriched GO terms related to “carbohydrate metabolism” and “localization” (Additional file 5: Table S3).

### Abundance of mitochondrial transcripts during early meiosis

**Figure 3:**
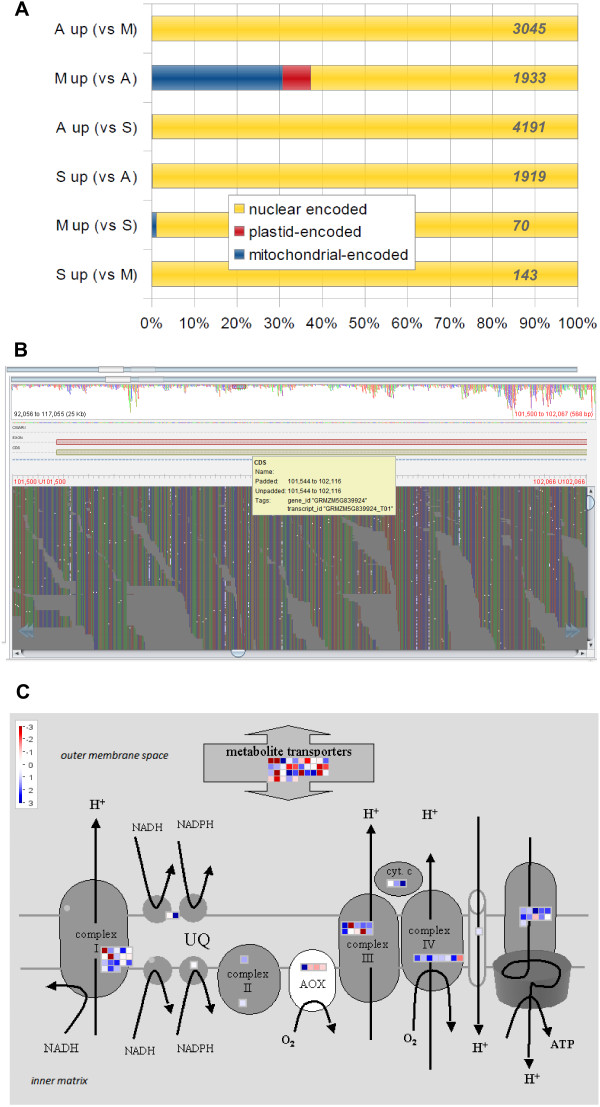
**Mitochondrial genes and RNA editing. (A)** Percentage of encoding locations of differentially expressed genes. Total number of genes per list in grey italic letters on the bars, A = anthers, M = meiocytes, S = seedlings. **(B)** Example for editing of mitochondrial transcripts. Read alignments to the genome reference, highlighting SNPs/edited sites in white. **(C)** Genes encoding components of the mitochondrial electron transport chain in meiocytes compared to seedlings. Scale shows a log_2_fold change between samples, blue = higher in meiocytes, red = lower in meiocytes.

We detected a large number of mitochondrial-functioning genes as highly expressed in isolated meiocytes vs. both anthers and seedlings (Figure 3A, Additional file 4: Figure S2B and Additional file 7: Figure S4). These transcripts originated from genes present in the nuclear genome as well as the mitochondrial genome. 24 out of the 69 genes up-regulated in meiocytes vs. anthers were mitochondrial-encoded, which is a significant proportion considering there are only 58 identified genes in mitochondria in maize [29] vs 39,656 genes in total (filtered gene set, version 2). In support of the mitochondrial origin of these transcripts, a close examination of mitochondrial transcripts in our dataset revealed C → U RNA editing (Figure 3B). Via a comprehensive SNP analysis on the mitochondrial chromosome, we detected G → A and C → T transitions, which both translate into C → U RNA editing, differing only in their strand origin (forward vs. complementary). We carried out a refined approach which only targeted C → U conversions in annotated genes (123 genes, including the 58 described genes plus novel ORFs, and 1 pseudogene) and found that up to ~2% of C’s were edited (Additional file 8: Table S4).

35 out of 59 nuclear- and mitochondrial-encoded genes for components of the mitochondrial electron chain were up-regulated in meiocytes compared to seedlings while only 9 were down-regulated, 3 of those encoding alternative oxidase (Figure 3C, also see Additional file 9: Figure S5); genes encoding metabolite transporters did not show an expression bias in meiocytes vs. seedlings.

### Expression level of meiotic gene candidates

We generated a list of meiotic gene candidates in maize using a list of *Arabidopsis thaliana* genes known to be involved in meiosis compiled from data of [5] and [22]. To find homologs of these genes in maize, the Arabidopsis genes were submitted to a gene family search using Phytozome (http://www.phytozome.net[30]). Putative maize homologs were selected according to the presence of similar domains and further examined regarding their expression levels in the maize RNA-seq dataset. Of the 81 putative maize meiotic gene candidates some but not all were found to be highly up-regulated in meiocytes: 24 were expressed at least 5-fold higher in meiocytes than in seedlings, but only four genes were expressed at a level of 2-fold or greater in meiocytes vs. anthers (Additional file 10: Table S5, examples in Table 2). In general, good indicators for a meiotic gene candidate in our maize dataset are 1) at least 5-fold higher expression in meiocytes than in seedlings (M/S ≥ 5), or, as in Arabidopsis [5]*,* 2) at least 2-fold higher expression in meiocytes/ anthers vs. seedlings, while expression in anthers is less than 4-fold that of meiocytes (A/M < 4, if M/S and A/S both ≥ 2; true for 44 out of 81 candidate genes in the meiotic gene list, with a large overlap with the first criterion of M/S ≥ 5).

**Table 2:** Examples of meiotic gene candidates

**Meiotic gene in Arabidopsis**	**Candidate in maize**	**Reads per million reads (RPM)**		
		**A**	**M**	**S**
*ASY1*	*GRMZM2G035996*^a^	224.79	225.15	25.25
*BRCA2a + b*	*GRMZM2G134694*	4.83	1.90	10.60
*BUB3.1*	*GRMZM5G899300 (= ZmBub3)*	112.14	146.32	36.08
*BUBR1*	*GRMZM2G009913*	44.42	54.51	13.33
*COM1 = GR1*	*GRMZM2G076617*^*a*^	9.64	13.41	2.53
*DIF1 = SYN1 = REC8*	*GRMZM2G059037*^bc^	170.46	346.00	15.55
*DMC1*	*GRMZM2G109618 (= ZmDmc1)*^b^	257.06	463.66	0.39
*DUET = MMD1*	*GRMZM2G408897*	6.37	5.88	1.54
*HOP2*	*GRMZM2G451604*	22.57	23.23	9.40
*MAD2*	*GRMZM2G047143 (= ZmMad2)*	45.46	65.01	21.85
*MER3 = RCK*	*GRMZM2G346278*^b^	128.24	116.04	3.02
*MLH3*	*GRMZM2G315902*	3.95	7.14	1.58
*MND1*	*GRMZM2G102242*^bc^	75.45	151.68	9.72
*MPS1 = PRD2*	*GRMZM2G133969*	36.74	40.14	11.79
*MRE11*	*GRMZM2G106056 (= ZmMre11A)*	103.42	117.33	39.45
*MSH2*	*GRMZM2G056075*^a^	45.73	86.59	17.00
*MSH4*	*GRMZM2G173186*	14.67	12.69	5.27
*MUS81*	*GRMZM2G361501*	9.29	3.46	5.35
	*GRMZM5G822970*^a^	42.62	71.29	7.21
*NBS1*	*GRMZM2G006246*	15.73	17.35	17.10
*OSD1*	*GRMZM2G089517*	10.84	13.85	6.74
*PHS1*	*GRMZM2G100103 (= ZmPhs1)*^b^	79.05	149.10	1.15
*PMS1*	*GRMZM2G058441*	7.58	9.63	2.60
*PRD3*	*GRMZM2G458423*^b^	16.28	34.08	0.95
	*GRMZM2G055899*	3.90	3.64	1.60
*PS1*	*GRMZM2G133716*	72.95	57.52	17.72
*PTD*	*GRMZM2G338661*^b^	33.81	37.24	0.10
*RAD50*	*GRMZM2G030128*	33.21	44.99	14.09
*RAD51*	*GRMZM2G055464 (= ZmRad51D)*^a^	21.08	22.65	4.04
	*GRMZM2G084762 (= ZmRad51A2)*	14.69	10.48	8.95
	*GRMZM2G121543 (= ZmRad51A1)*	21.37	11.37	9.28
*RAD51C*	*GRMZM2G123089*	14.47	19.12	8.97
*RMI1*	*GRMZM2G108255*^a^	41.27	68.42	9.78
*SCC2*	*GRMZM2G132504*	81.01	85.84	36.95
*SDS*	*GRMZM2G344416*^b^	10.73	13.21	0.03
	*GRMZM2G093157*^a^	29.82	42.90	3.34
*SMC6*	*GRMZM2G025340*	37.77	65.07	29.46
*SPO11-1*	*GRMZM2G129913*^ac^	15.72	31.38	4.04
*SPO11-2*	*GRMZM5G890820*^a^	8.81	9.85	1.30
*SRP2*	*GRMZM2G440605*	0.19	0.28	0.06
*SWI1 = DYAD*	*GRMZM2G300786*^b^	4.84	7.46	0.11
	*GRMZM5G883855 (= ZmAm1)*^b^	23.89	17.74	0.25
*TOP3A*	*GRMZM2G470438*	40.68	43.95	17.45
*XRCC3*	*GRMZM2G157817*	4.81	7.28	2.20
*XRI1*	*GRMZM2G091168*	21.84	26.56	6.65
*ZIP4*	*GRMZM2G064382*	12.38	17.50	8.15
*ZYP1a + b*	*GRMZM2G143590*^bc^	43.74	106.33	9.61

^a^S/M ratio > 5, ^b^S/M ratio > 10, ^c^M/A ratio > 2.

*Mus81* is an example of employing these criteria to meiotic function candidate genes to support the selection of the best candidates for function in meiosis: *GRMZM2G361501* has the lowest expression in the meiocytes sample, while *GRMZM5G822970* has an almost 10-fold ratio in meiocytes vs. seedlings and an almost 2-fold ratio in meiocytes vs. anthers. Other examples, such as *Rad51D* show a pronounced increase in expression in meiocytes vs. seedlings, and *Rad51A1* and *Rad51A2*[31] are also highly expressed in meiocytes vs. seedlings, but to a lesser extent. Detection of *Rad51A1* and *Rad51A2* confirms the feasibility of the approach using Phytozome, though not everything can be detected, e.g. for the Arabidopsis *SWI1/DYAD* gene the search found the maize paralog *Am1* but not *AC194609.2_FG029* although they stem from a duplication event [32].

### Validation of gene expression and its importance in meiocytes by RNA in situ hybridization, real-time RT PCR and in silico analysis

**Figure 4:**
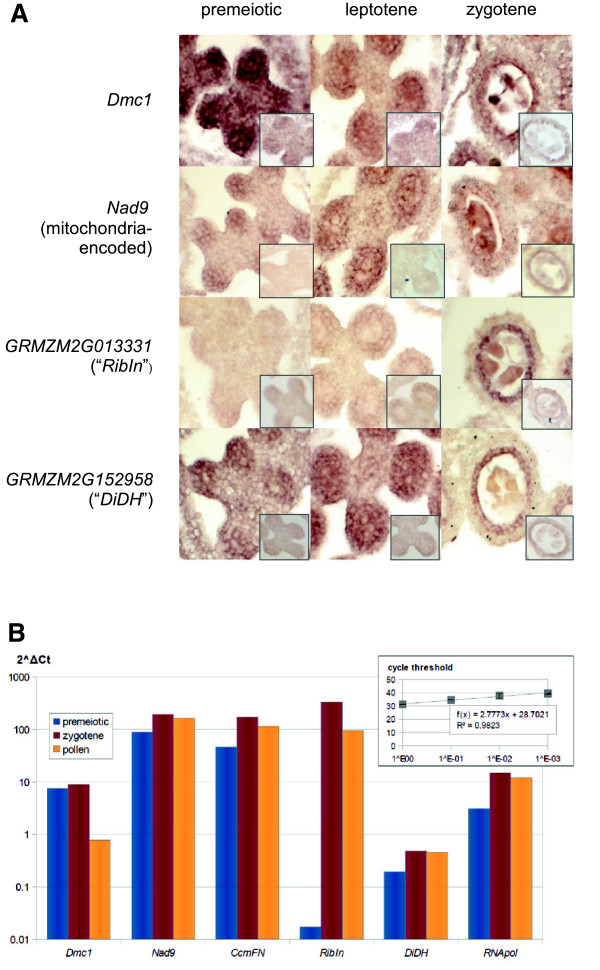
**RNA *in situ *hybridization and real-time RT PCR. (A)** RNA *in situ* hybridization on cross sections of anthers from various anther development stages shows the locations of RNA transcripts. Signals ranged from very strong (*Dmc1* premeiotic) to non-existent (*GRMZM2G013331* premeiotic). Smaller inserts in the panel show controls (sense probes). **(B)** Real-time RT-PCR analysis of RNA from whole anthers at the premeiotic, zygotene and pollen stages. Expression level normalized with a reference gene, HMG (*GRMZM5G834758*), depicted as 2^ΔCt^. *Dmc1* = coding for meiotic recombinase DMC1; *Nad9* = coding for subunit of NADH-dehydrogenase, mitochondrial-encoded; *RibIn* = coding for putative ribosome-inactivating protein; *DiDH* = coding for putative dihydrolipoyl-dehydrogenase; *CcmFN* = coding for component of cytochrome C biogenesis, mitochondrial-encoded; *RNApol* = coding for putative RNA polymerase, mitochondrial-encoded.

A previous similar approach in Arabidopsis also identified genes important for meiosis [5], and was followed up by a promoter study to prove the meiocyte-specific expression of candidate genes [33]. Here, we choose multiple approaches to verify the gene expression patterns detected in the RNA-seq data. To extend the analysis to detection of tissue specificity we selected several genes for further analyses using RNA *in situ* hybridization and real-time RT-PCR. The example genes we chose contained a well-known meiosis gene as a positive control, mitochondrial-encoded genes which have been found to be of interest in this study, and genes expressed at low levels in order to ascertain their expression pattern. The genes selected for RNA *in situ* hybridization included *Dmc1* (known to be critical for meiotic recombination [34,35], highly expressed in meiocytes), *Nad9* (mitochondrial-encoded, component of complex I in the mitochondrial electron transport chain, highly expressed in meiocytes), *GRMZM2G013331* (encoding an uncharacterized ribosome-inactivating protein, expressed at low levels) and *GRMZM2G152958* (encoding an uncharacterized dihydrolipoyl-dehydrogenase, expressed at low levels). RNA *in situ* hybridization results for *Dmc1* and *Nad9* indeed showed that both genes were strongly expressed in anther lobes (Figure 4A, Additional file 11: Figure S6). The signals were especially strong in premeiotic and leptotene anthers, and were concentrated in areas where the meiocytes develop, but not in the connective tissue between anther lobes. The occurrence of *Dmc1* expression before the onset of meiosis has been reported before, for example in wheat [14]. In zygotene, the signals were more confined to meiocytes. *Dmc1* was also expressed in the tapetum (Figure 4A).

RNA *in situ* hybridization with *GRMZM2G013331* and *GRMZM2G152958* showed expression in tapetum cells in zygotene anthers. In addition, *GRMZM2G152958* was expressed throughout anther lobes in premeiotic and leptotene anthers, comparable in strength with *Dmc1* and *Nad9* (Figure 4A). However, *in situ* hybridization is better used as a relative quantitative method for comparing tissues in a sample, but might not be the tool of choice to compare between the expression strength of different genes due to e.g., hybridization strength differences (which is also a drawback of microarray experiments). Real-time PCR and RNA-seq data are usually in good agreement and can help to decide if unique or apportioned counts better reflect the actual expression levels (see Additional file 12: Table S6).

To verify expression levels of the selected genes, we also performed real-time PCR of cDNAs from whole anthers at premeiotic, zygotene and pollen stages (Figure 4B). Samples included the four genes examined with *in situ* hybridization and two additional mitochondrial-encoded genes, *CcmFN* (*GRMZM5G867512*, coding for a component of the cytochrome C biogenesis pathway) and *RNApol* (*GRMZM5G827309*, coding for a putative DNA-dependent RNA polymerase). The results were similar to those obtained from *in situ* hybridization, including strong *Dmc1* expression in early stages and almost undetectable level of *GRMZM2G013331* in premeiotic-stage anthers (see also Additional file 11: Figure S6, Additional file 12: Table S6).

To verify not only the expression of genes detected as preferentially expressed in meiocytes but also their importance, we analyzed the generated gene list for the presence of named maize genes, interpro descriptions and meiosis candidate genes (Additional file 3: Table S2). Twenty of our meiosis-candidate genes were contained in the list, most notably *Am1* and *Phs1* which have already been shown in maize to be involved in meiotic recombination and whose loss results in male sterility [16,32,36].

## Discussion

Previous studies have addressed the important question of the meiotic transcriptome in plants: Microarray-based approaches in maize, petunia, wheat and rice [10,13-15] examined different developmental stages of anthers and provided valuable information on transcriptome dynamics and genes specific to certain stages. These and other studies aided in identifying meiotic genes in Arabidopsis, maize, barley and rice. Here we complement these efforts by providing a comprehensive atlas of meiotic gene candidates in maize, together with the expression level in isolated early meiocytes. Another goal of our current study was to take advantage of our data from isolated meiocytes, to detect processes vital in early meiosis as indicated by transcript abundance. By sequencing the transcriptome of isolated maize meiocytes at leptotene and zygotene, we generated an expression profile of early meiotic prophase I in plants.

Isolated meiocytes and whole anthers had very similar expression profiles, which is not surprising since whole anthers contain meiocytes. A previous study suggested that maize meiocyte RNA contributes up to 20% of whole anther RNA [16]. They based their calculation on data from rice [8] and Arabidopsis [5], estimating 25 times (rice PMCs) and 100 times (Arabidopsis meiocytes) more RNA than in typical diploid cells [16]. In maize, PMCs constitute less than 1% of the total anther cells but because they are large (around 10% of the anther volume) [16], the use of whole anthers to obtain information about meiotic transcriptomes had been justified. Our data now unveils that the contribution of meiocytes to the whole anther transcriptome landscape might be far greater than previously assumed, at least in maize. With this in mind, previous transcriptome anther data can be reanalyzed, and future RNA level studies aiming at the meiotic transcriptome can reasonably use whole anthers instead of isolated meiocytes. Nevertheless, using isolated meiocytes yielded unprecedented resolution and novel insights into specific expression patterns. Furthermore, for DNA-based experiments, the contribution of meiocytes to whole anthers is far lower and the use of meiocytes is strongly suggested.

### Abundance of mitochondrial transcripts in meiocytes

Unlike Arabidopsis in which significant differences in the gene expression landscape were detected between meiocytes and anthers [5], isolated meiocytes and whole anthers of maize had very similar expression profiles. We detected a significantly increased amount of mitochondrial transcripts in early prophase I meiocytes, which would not have been obvious from whole-anther data. Organelle transcripts have been encountered in other transcriptome studies before [37] but are usually dismissed without further explanation, or regarded as artifacts [27]. According to the classical view, polyA selection during library preparation should indeed remove most mitochondrial mRNAs. However, the classical view of stabilizing 3′polyA only on transcripts from the nucleus and transient degradation-targeting external and internal polyA on transcripts in bacteria and organelles was recently challenged by diverse studies [37-39]. A strong increase of poly-adenylated transcripts from mitochondrial genes was detected in recent Arabidopsis studies connected with enhanced thermotolerance [37,40,41] and some plant studies on development have encountered elevated transcript levels of specific mitochondrial genes [42-46]. Explanations range from gene dosage effect to transcriptional activation to higher stabilization, but the molecular basis and significance are not elucidated.

In plants, there is an ongoing shuffling of mitochondrial genome segments to the nucleus, leading to NUMTs (**nu**clear encoded **m**i**t**ochondrial DNA), which can occur via insertion of the entire or parts of the mitochondrial genome (DNA) or of processed transcripts (mRNA) [47-50].

The question has to be posed whether the mitochondrial gene transcripts detected in our RNA-seq arose from NUMTs (also called MGI, **m**itochondrial **g**enome **i**nsertion) or directly from the mitochondria. We detected a high proportion of edited mitochondrial transcripts in our RNA-seq data which points to mitochondrial origin. Approximately one-third of the genes that are highly expressed in meiocytes vs. anthers are encoded in mitochondria, but nuclear-encoded genes with functions in mitochondria are also up-regulated in meiocytes vs. seedlings. We hypothesize that this is an indication of a high energy demand during early prophase I, when vigorous chromosome movement occurs [51]. Consequently, our data indicate that genes encoding proteins involved in the glycolysis step and the mitochondrial electron transport chain show significantly increased expression levels in meiocytes. A few studies from other organisms also point to the importance of mitochondrial processes for the meiotic pairing process, namely in *Caenorhabditis elegans* and *Schizosaccharomyces pombe* where chromosome pairing or recombination was dependent on mitochondrial respiration [52,53]. The functional requirement of mitochondrial genes for meiosis or post-meiotic processes is highlighted by the phenomenon of cytoplasmic male sterility, which is due to mutated mitochondrial genes and has been reported in maize and other plants [54-59]. Despite those clues for the importance of mitochondria-located processes for meiosis, both previous Arabidopsis meiocyte transcriptome studies had not pointed this out. Not explicitly mentioning any mitochondria genes up-regulated in meiocytes, Yang *et al*. [22] still listed the 18 most enriched PFAM families including Mito-Carr (Mitochondrial carrier), and TPR_1 and TPR_2 (Tetratricopeptide repeats) which can be found in the NADPH oxidase subunit and as receptor of mitochondrial import proteins. Chen *et al.*[5] noted the increase of transcripts from mitochondria origins, but attributed it to a mitochondrial genome insertion (MGI) because the reads mapped to this region. A new analysis, comparing *Arabidopsis* and maize data generated in our lab recently revealed that the abundant transcripts in *Arabidopsis* also originated directly from the mitochondrial genome [24].

### Redox homeostasis and chromatin modification during early meiosis

Two groups of genes that are highly expressed in meiocytes also deserve closer attention. One is “homeostasis", encompassing genes encoding thioredoxins and glutaredoxins. The redox status has recently been postulated to be a determinant of cell fate in pre-meiotic anther development [60]. A central key player in establishing germ cell initiation is *Msca1 (****M*** *ale* ***s*** *terile* ***c*** *onverted* ***a*** *nther* ***1****)*[60], which encodes a glutaredoxin and is also on our list of “homeostasis” genes whose expression is up-regulated in early meiosis, together with two other putative glutaredoxins and five thioredoxins (Additional file 5: Table S3). Thioredoxins are ubiquitous disulfide regulatory proteins that seem to link redox status to cell fate and growth during development in multicellular organisms [61,62] and in Arabidopsis, thioredoxin Trx H9 even appears to be capable of cell-cell-migration and communication [63]. The detected redox regulator candidates might not only be required for establishment of germ cells but also for maintaining, progressing and especially synchronizing the meiotic process later on.

The other enriched process group is “chromatin”, indicating that expression of chromatin-related genes may represent a link to meiotic recombination. For example, histone 3 lysine 4 trimethylation (H3K4me3) marks double strand break hotspots in mouse [64], and histone acetylation is often linked with recombination-active regions [65-67]. Our data provides a list of candidates for meiosis-specific histone modifiers that might influence recombination, including two SET domain proteins and two proteins from the histone deacetylase superfamily (Additional file 5: Table S3).

### Protein localization and degradation

We also found processes connected with molecule targeting, localization and proteolysis significantly enriched in meiocytes. While some genes implicated in localization processes might be due to the abundant mitochondrial transcripts, there is also a connection with proteolysis-related components. Ubiquitination is mostly associated with proteolysis, has diverse roles in plants and is important for regulating growth and development, reviewed in [68,69], with a non-degradative function in regulating cellular localization and activity, reviewed in [70]. Monoubiquitination is implicated in regulation of membrane transport and transcriptional processes [71], making the genes up-regulated in meiocytes involved in these processes also interesting candidates for further studies.

## Conclusions

The generated early meiosis-specific transcriptome dataset of maize is a valuable resource for understanding the meiotic program. Using it, we were able to reveal novel and under-acknowledged aspects of early meiosis such as high energy production, ER-connected processes, and RNA regulation. In addition to identifying new meiotic gene candidates, this dataset can be useful for distinguishing between genes functioning in meiocytes vs. other tissues of the meiotic anther to provide insight into the interaction between meiocytes and somatic cells of the anther. Taken together, future studies could aim to investigate how the processes detected herein are connected and which regulatory roles they play to direct events during early meiosis.

## Methods

### Plant material and isolation of maize male meiocytes

Plants of the maize *(Zea mays)* B73 inbred were grown in the greenhouse, in 16 hours of light (~450 μmol × m^-2^ × sec^-1^) at 24°C and 8 hours of darkness at 22°C, in a mix of top soil and SunGro LC8 (2:1), and were fertilized with ~30 g slow-release fertilizer (Osmocote 14-14-14) and biweekly addition of ~1-2 g of Peterson’s 20:20:20 dissolved in water. To determine the meiotic stage, we used acetocarmine staining as described in Sheehan and Pawlowski (2012), intensified by use of ferric oxide. We collected only meiocytes and anthers of early prophase I (leptotene and zygotene), storing them in RNA*later*. The anthers that were used for the experiments came from the same plants that were used for meiocyte collection (~10-15 plants per replicate). Isolation of plant meiocytes followed the general procedure for CCM (**C**apillary **C**ollection of **M**eiocytes) described for Arabidopsis [5,20,22] and maize [72], handling freshly plucked spikelets for no longer than 20–30 minutes before immersing the isolated cells into RNA*later*. In the case of maize, anthers with early meiotic stages (leptotene and zygotene) were dissected and squashed with a flattened pipet tip. On average, ~800 cells were collected per hour and around 20,000-30,000 cells were used for RNA extraction (for RNA yield, see Additional file 1: Table S1). For each seedling replicate, three approximately two-week old seedlings with three leaves were used. All biological replicates were sampled at independent times in the greenhouse.

### RNA extraction and sequencing

The Ambion RNAqueous Micro Kit (Ambion) was used to extract total RNA from meiocytes, whole anthers and seedlings. The RNA amount was measured using the Qubit® RNA BR Assay Kit with the Qubit® Fluorometer (Invitrogen).

RNA libraries were prepared using standard Illumina TruSeq RNA library kits which select for polyA RNAs. Libraries were sequenced on Illumina HiSeq 2000 instruments to generate single-end 50 nt-long reads. Sequences were filtered with standard Illumina pipelines and additional filtering steps were used to identify adapters (Additional file 13: Table S7).

### Transcriptome analysis

Reads for each sample were aligned to the maize B73 genome reference (RefGenv2, annotation release 5b.60) with GSNAP v 2011_03_28 [73] with default parameters except max-mismatches = 2, indel-penalty = 2, novelsplicing = 1, localsplicedist = 1000, distantsplicepenalty = 1000, terminal-penalty = 1000, npaths = 10, and known splice sites were fed into the alignments. Gene read counts were generated using a pipeline developed at NCGR [74]. Only the best match(es) for a given read were considered. Reads were assigned to a gene if it mapped to the coordinates of the gene, including reads that overlapped but were not completely contained within the gene. Alternative splice isoforms were not considered individually. For unique read counts, used in gene analyses, only reads with a single best hit were considered. For TE analysis and for analyses comparing genes and TEs, GSNAP alignments were rerun with the same parameters except that up to 100 paths were returned (npaths = 100). As before, only the best alignment(s) were considered. Rather than only considering reads that align uniquely, we accounted for ambiguously mapped reads (reads mapping equally well to multiple genes) by distributing them proportionally to all possible genes. Reads were assigned to genes in a hierarchical fashion beginning with reads with only a single match, followed by reads with two matches, etc. Accounting for alignment ambiguity was important for TEs because they are repetitive by nature. Indeed, there was nearly a three-fold increase in the number of TEs showing expression with these criteria compared to using only unique hits. Gene-based analyses changed minimally with the two criteria sets (< 4% increase in the number of genes expressed in each replicate with an average of 1.6%). Therefore, to be able to compare directly between genes and TEs in analyses that involved both genes and TEs the apportioned read data were used.

For the gene-based differential expression analyses, unique count data were used. After removal of all genes showing zero expression, only the top 70 percent of the normalized data were taken into account for most analyses, including detection of differentially expressed genes, similar to the cutoff suggested by [75]. Pearson’s correlation coefficients differed only slightly when considering all unique counts instead of the trimmed set (anthers: 0.9419 trimmed/0.9438 all; meiocytes: 0.9174 trimmed/0.9187 all; seedlings: 0.7990 trimmed/0.8031 all). With JMP® Genomics (Version 6.0, 64-bit Edition), hierarchical clustering on correlations between the biological replicates was tested with the Ward method. The analysis to detect differentially expressed genes was performed with the DEseq package for R [76] openly available as part of the Bioconductor suite of R tools. The DEseq package was used with the trimmed data for MA ratio calculation and plotting, as well as for the generation of lists of genes exhibiting significant differential expression among samples with a P value adjusted after Benjamini-Hochberg correction for multiple testing ≤ 0.01 [77]. Similar results were obtained when using lists of differentially expressed genes with an adjusted *P* value ≤ 0.05. Venn diagrams were calculated and depicted with JMP Genomics, using either the complete data normalized to read per million reads (RPM) or the lists of differentially expressed genes generated with R. Beside using R-generated lists without modification, further meiosis-gene definition (M/A > 2, or A/M < 4, if M/S and A/S both ≥ 2) was applied on a combined list of differentially expressed genes up-regulated in anthers or meiocytes vs. seedlings.

For GO (gene ontology) analysis, the lists of differentially expressed genes were submitted to the AgriGO GO tool [28], http://bioinfo.cau.edu.cn/agriGO/ for Singular Enrichment Analysis with the default setting for Fisher’s exact test adjusted for multiple testing with the Benjamini-Yekutieli method and to MapMan [27].

For TE analysis, a modified version of the file ZmB73_5a_MTEC_repeats.gff (maizesequence.org) where duplicates had been removed was used to generate read counts for the annotated ~1.7 million TE locations. TEs with less than or equal to five reads total in our three tissue sources were excluded. This equals a 23.5% upper cut instead of the 70% used with the gene data set. Downstream analyses were performed with lists of differentially expressed TEs generated with DEseq, and with a list of TEs designated as meiosis-specific. For the latter one, we used only highly expressed TEs (row sum > 1000), and applied the same definition criteria as for genes (M/A > 2, or A/M < 4, if M/S and A/S both ≥ 2), and used the remaining TEs for comparison.

Visualization of aligned reads to the genome was performed using Tablet Viewer Version 1.12.08.29 [78], with reference gene features loaded for comparison. Tablet Viewer was also used for detection and verification of RNA editing in mitochondrial genes.

### RNA in situ hybridization

Primers were designed for cloning the complete CDS into the pGEM-Teasy vector for probe synthesis with RNA polymerases T7 and SP6 using the DIG RNA labeling kit (Roche). The RNA *in situ* hybridization procedure closely followed the protocol described in Yong et al. (2003) and was performed with minor modifications. Whole maize tassels were fixed for 1 hour under vacuum on ice and then kept in the fixative overnight at 4°C. Instead of xylene, histo-clear was used for clearing, and before the detection reaction, slides were RNAse treated and washed with a low-stringency buffer (2× SSC). 3 hours of developing in the dark were used for all probes and gave sufficient signals.

### Real-time RT PCR

B73 maize anthers were staged, dissected and stored in RNA*later*. Immediately before processing, anthers were washed in 1xPBS and then used for RNA extraction using the Qiagen RNeasy® Mini Kit. DNAse digest was carried out with Optizyme rDNAseI (Fisher Scientific). ~2.5 μg total RNA per sample as measured with Qubit® RNA BR Assay Kit (Invitrogen) was used for cDNA synthesis with the SuperScript® III First Strand Synthesis System for RT-PCR (Invitrogen) including oligo dT primer. For real-time PCR, iQ™ SYBR® Green Supermix (Bio-Rad) was used with cDNA from ~125 ng total RNA and 25 μl end volume per reaction, performed in triplicates. Primers used are listed in Additional file 12: Table S6. Primer efficiencies were calculated with Real-time PCR Miner [79], http://www.miner.ewindup.info/ and were in the range of 90-110% per gene. A Bio-Rad real-time PCR system (C100TM Thermal Cycler, with CFX96™ Real-Time System) was used in conjunction with the Bio-Rad CFX Manager™ Software, Version 2.0.

## Availability of supporting data

Raw Illumina reads have been deposited into NCBI’s SRA (sequence read archive) under the study title “mRNA-seq of *Zea mays* B73 early-prophase meiocytes, anthers, and seedling control”, accession numbers SRX218264-218270.

## Competing interests

The authors declare that they have no competing interests.

## Authors’ contributions

SD performed research, analyzed data and wrote the manuscript, AS, JM, TR, and AF, analyzed data and edited the manuscript, MW, QS and JP helped with data analysis and manuscript editing, SK, ER, WP and CC designed the research and edited the manuscript. All authors read and approved the final manuscript with the exception of Ernest Retzel, who sadly passed away.

## Supplementary Material

Additional file 1: Table S1 RNA yield, alignment scores of sequenced reads, and Pearson’s correlation coefficients between all RNA-seq. Click here for file

Additional file 2: Figure S1 Differentially expressed genes. Differentially expressed genes in meiocytes, anthers and seedlings of *Zea mays* B73. (A)-(C) MA plots of DE genes in meiocytes vs anthers (A), anthers vs seedlings (B), meiocytes vs seedlings (C). (D) Heatmap of combined DE genes up in anthers or meiocytes versus seedlings. Click here for file

Additional file 3: Table S2 Lists of differentially expressed genes. Click here for file

Additional file 4: Figure S2 Transposable elements. (A) Average proportion of global expression (apportioned reads, up to 100 equally-good matches). (B) Percentage of apportioned reads per feature for each sample. Y-axis scale is logarithmic. (C-F) Distribution analysis of different patterns in subsets of TEs: transposon order (C), transposon superfamily (D), chromosomal location of transposon (E), special families with a previously shown connection to meiotic or mitotic tissues (F). All subsets of differentially expressed genes are shown in (C), the subset defined as meiosis-specific is compared with the non-meiosis-specific subset in (C-F). Click here for file

Additional file 5: Table S3 GO terms in differentially expressed genes. Click here for file

Additional file 6: Figure S3 Localization and transcription factors. Analysis with MapMan. Scale shows log_2_fold change between samples, blue = higher in meiocytes, red = lower in meiocytes. (A) Genes in molecule targeting machinery between meiocytes and seedlings. (B) Genes for transcription factors between meiocytes and seedlings. Click here for file

Additional file 7: Figure S4 Cellular components enriched in meiocytes and anthers. (A) Graph of cellular components significantly up-regulated in meiocyte vs seedling (P_adj_ ≤ 0.01). (B) Graph of cellular components significantly up-regulated in anther vs seedling (P_adj_ ≤ 0.01). Click here for file

Additional file 8: Table S4 Mitochondrial RNA editing. Click here for file

Additional file 9: Figure S5 Details of the TCA cycle and electron transport chain. (A) Differences in TCA cycle between meiocytes and seedlings. (B) Differences in mitochondrial electron transport chain in detail, in genes defined as meiosis genes. Scale shows log_2_ fold change between samples, blue = higher in meiocytes, red = lower in meiocytes. Analysis done with MapMan. Click here for file

Additional file 10: Table S5 Complete list of meiotic gene candidates. Click here for file

Additional file 11: Figure S6 RNA in situ hybridization Original images of all stages, and corresponding RNA-seq counts. (A) Table of genes used for in situ hybridization and their RPM (reads per million) counts. (B) Original in situ hybridization images. The same microscope and camera settings were used for all pictures and no editing was conducted with the cropped pictures. Click here for file

Additional file 12: Table S6 Comparison of RNAseq, *in situ* hybridization and real-time PCR. Click here for file

Additional file 13: Table S7 Primer and adapter information. Click here for file
