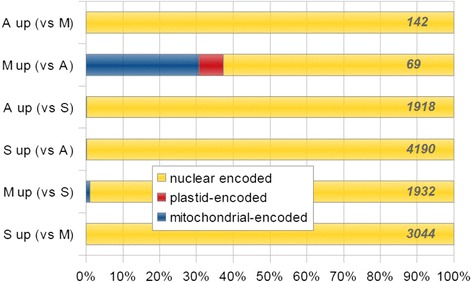# Correction to: The transcriptome landscape of early maize meiosis

**DOI:** 10.1186/s12870-017-1224-y

**Published:** 2018-01-15

**Authors:** Stefanie Dukowic-Schulze, Anitha Sundararajan, Joann Mudge, Thiruvarangan Ramaraj, Andrew D. Farmer, Minghui Wang, Qi Sun, Jaroslaw Pillardy, Shahryar Kianian, Ernest F. Retzel, Wojciech P. Pawlowski, Changbin Chen

**Affiliations:** 1 Department of Horticultural Science, University of Minnesota, St. Paul, MN 55108 USA; 2 National Center for Genome Resources, Santa Fe, NM 87505 USA; 3 Department of Plant Breeding and Genetics, Cornell University, Ithaca, NY 14850 USA; 4 Computational Biology Service Unit, Cornell University, Ithaca, NY 14850 USA; 5 USDA-ARS Cereal Disease Laboratory, University of Minnesota, St. Paul, MN 55108 USA

## Correction

Following publication of the original article [1], the authors reported that the number of genes overlaying the bar graph in Fig. 3A were incorrectly counted and inserted (i.e. including a title tile, and in inverse order). The corrected numbers are below and match with the listed genes supplied in Additional File: Table S2.

Previous, published version of Fig. 3A:

**Figure:**
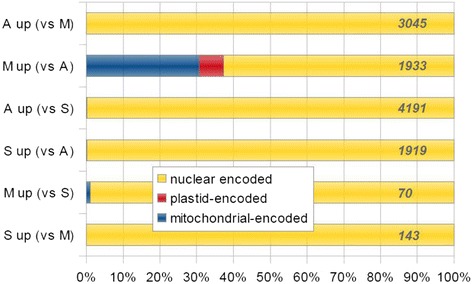

New, corrected version of Fig. 3A: