# Supplementary figures and images for: The transcriptome landscape of early maize meiosis
Source: BMC Plant Biol. 2014 May 3;14:118. doi: 10.1186/1471-2229-14-118 (PMC4032173; doi:10.1186/1471-2229-14-118)

# Supplemental Figure 1

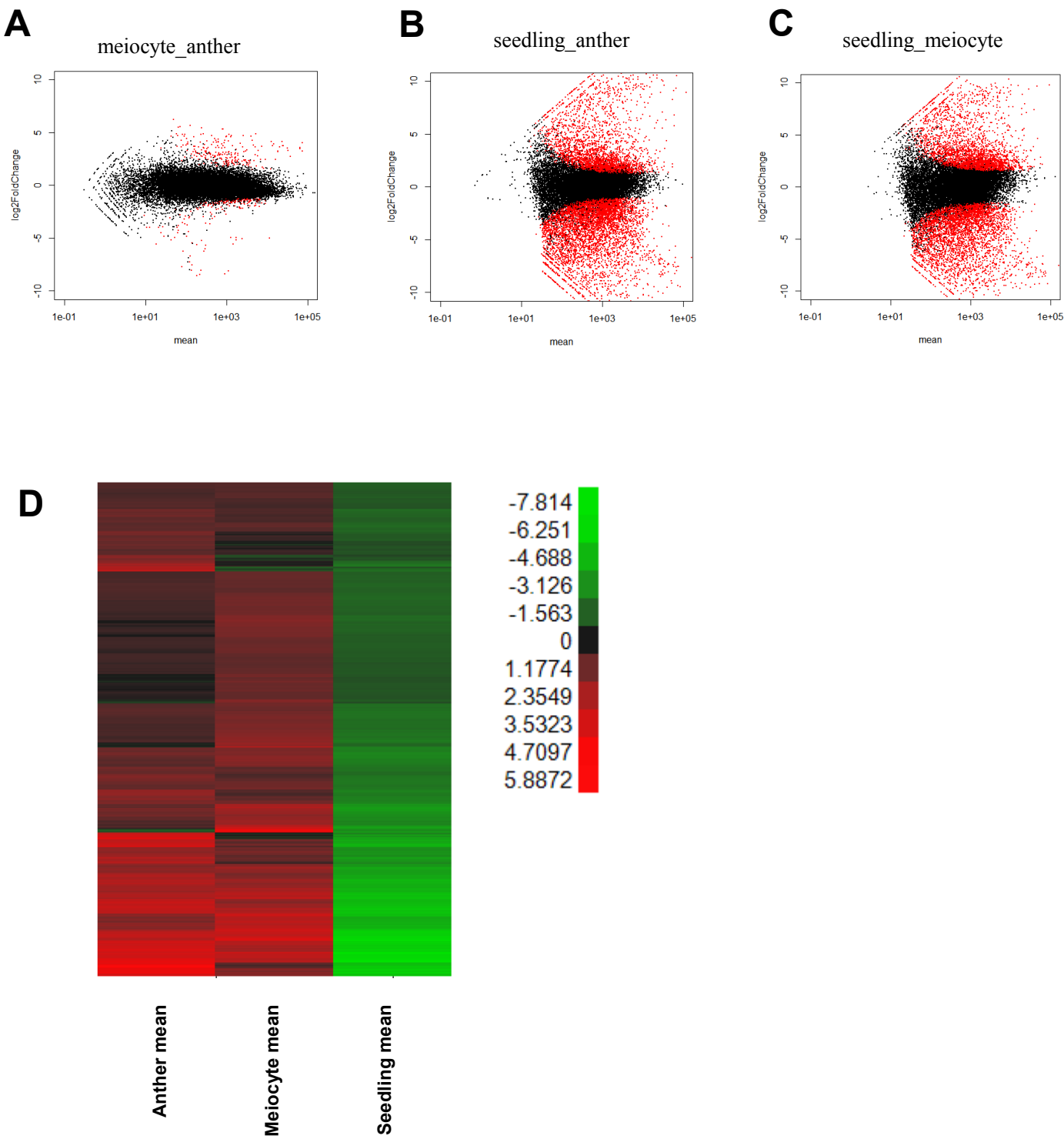

Supplement: Additional file 2: Figure S1 — Differentially expressed genes. Differentially expressed genes in meiocytes, anthers and seedlings of Zea mays B73. (A)-(C) MA plots of DE genes in meiocytes vs anthers (A), anthers vs seedlings (B), meiocytes vs seedlings (C). (D) Heatmap of combined DE genes up in anthers or meiocytes versus seedlings. [file 1471-2229-14-118-S2.pdf]

# Supplemental Figure 2

A

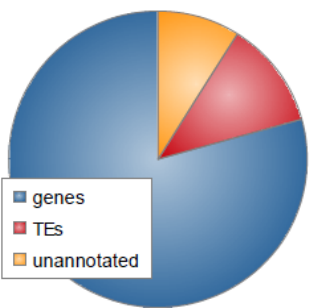

B proportion of apportioned reads [%]

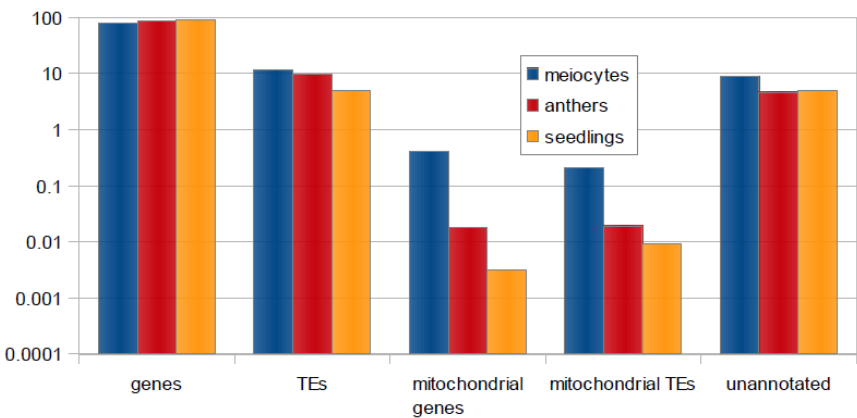

C

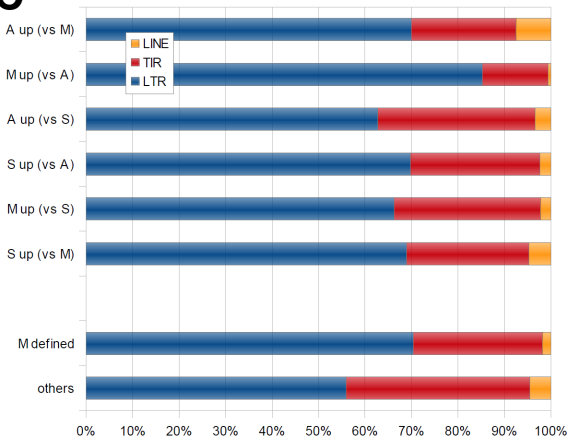

D

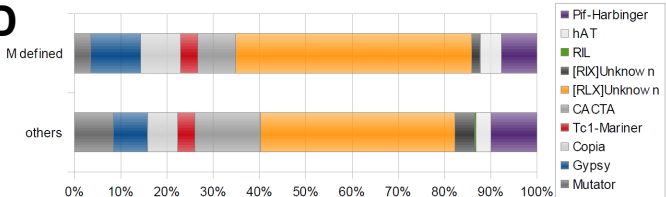

E

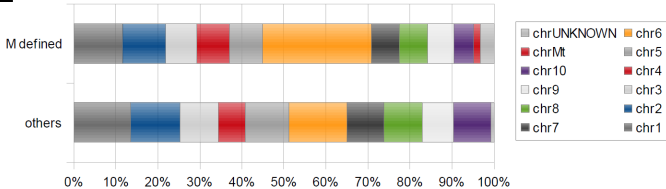

F

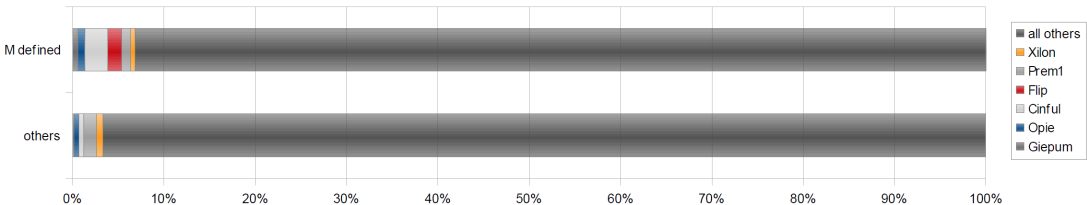

Supplement: Additional file 4: Figure S2 — Transposable elements. (A) Average proportion of global expression (apportioned reads, up to 100 equally-good matches). (B) Percentage of apportioned reads per feature for each sample. Y-axis scale is logarithmic. (C-F) Distribution analysis of different patterns in subsets of TEs: transposon order (C), transposon superfamily (D), chromosomal location of transposon (E), special families with a previously shown connection to meiotic or mitotic tissues (F). All subsets of differentially expressed genes are shown in (C), the subset defined as meiosis-specific is compared with the non-meiosis-specific subset in (C-F). [file 1471-2229-14-118-S4.pdf]

Supplemental Figure 3

A

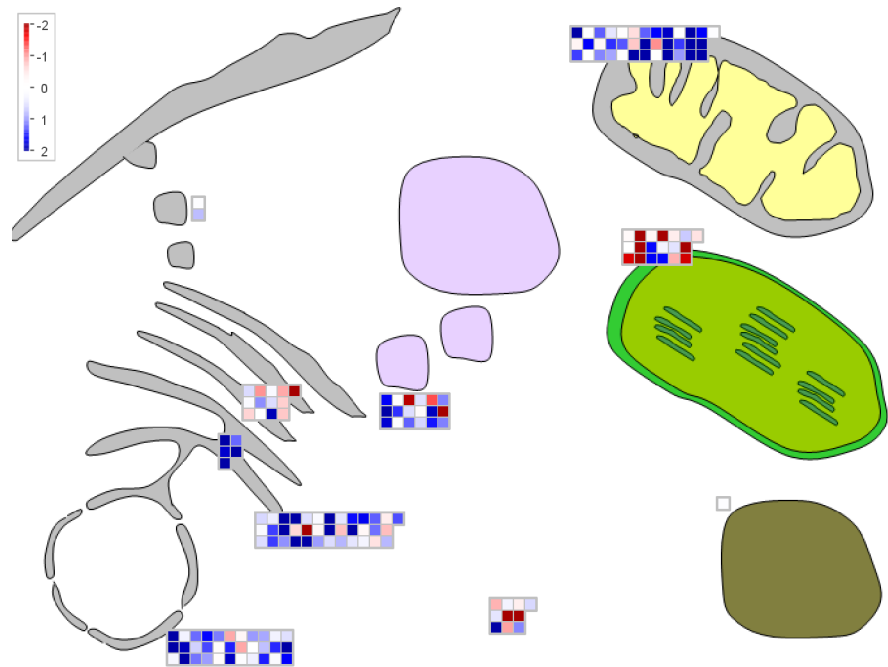

B

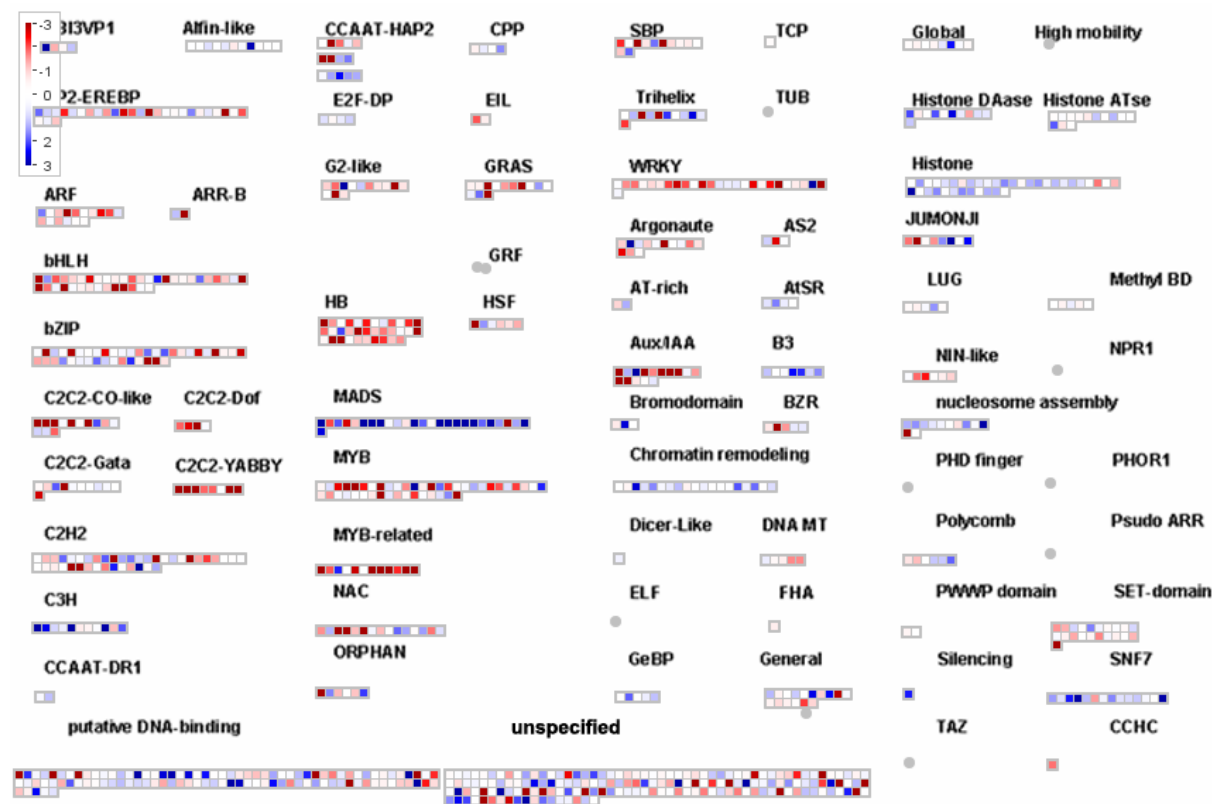

Supplement: Additional file 6: Figure S3 — Localization and transcription factors. Analysis with MapMan. Scale shows log2fold change between samples, blue = higher in meiocytes, red = lower in meiocytes. (A) Genes in molecule targeting machinery between meiocytes and seedlings. (B) Genes for transcription factors between meiocytes and seedlings. [file 1471-2229-14-118-S6.pdf]

## Supplemental Figure 4

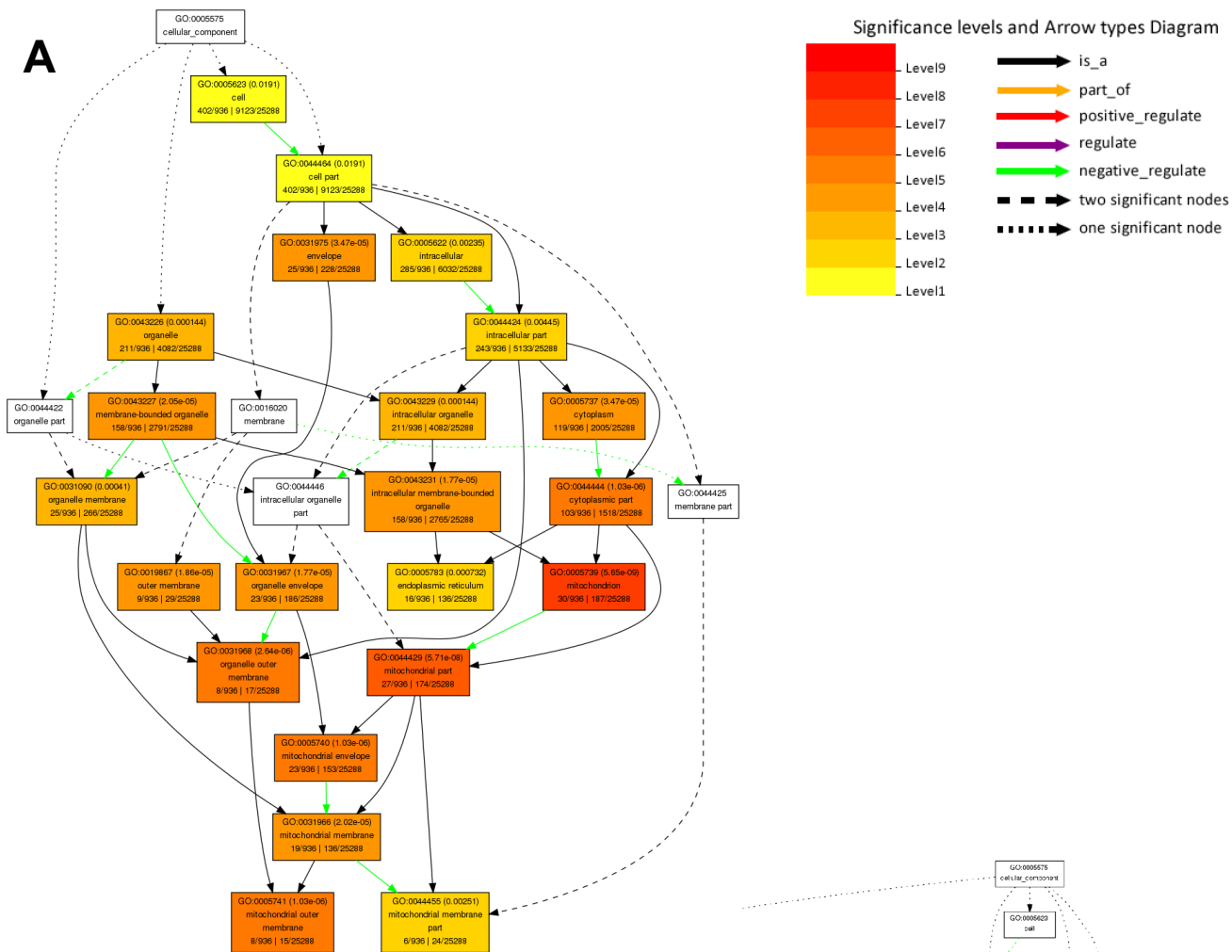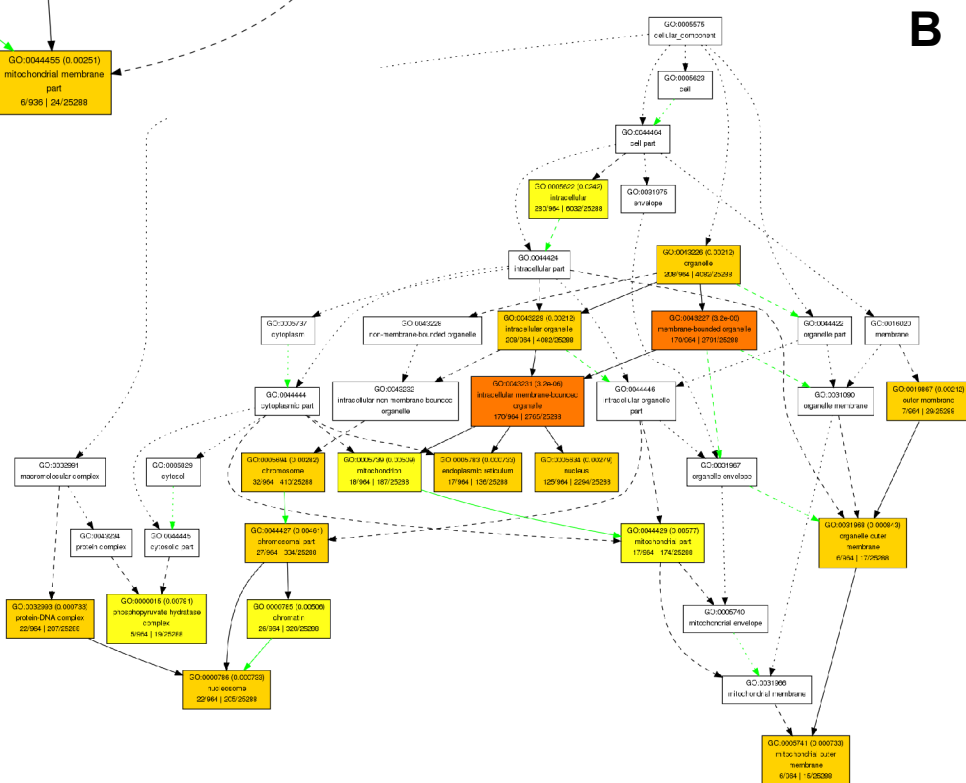

Supplement: Additional file 7: Figure S4 — Cellular components enriched in meiocytes and anthers. (A) Graph of cellular components significantly up-regulated in meiocyte vs seedling (Padj ≤ 0.01). (B) Graph of cellular components significantly up-regulated in anther vs seedling (Padj ≤ 0.01). [file 1471-2229-14-118-S7.pdf]

## Supplemental Figure 5

**A**

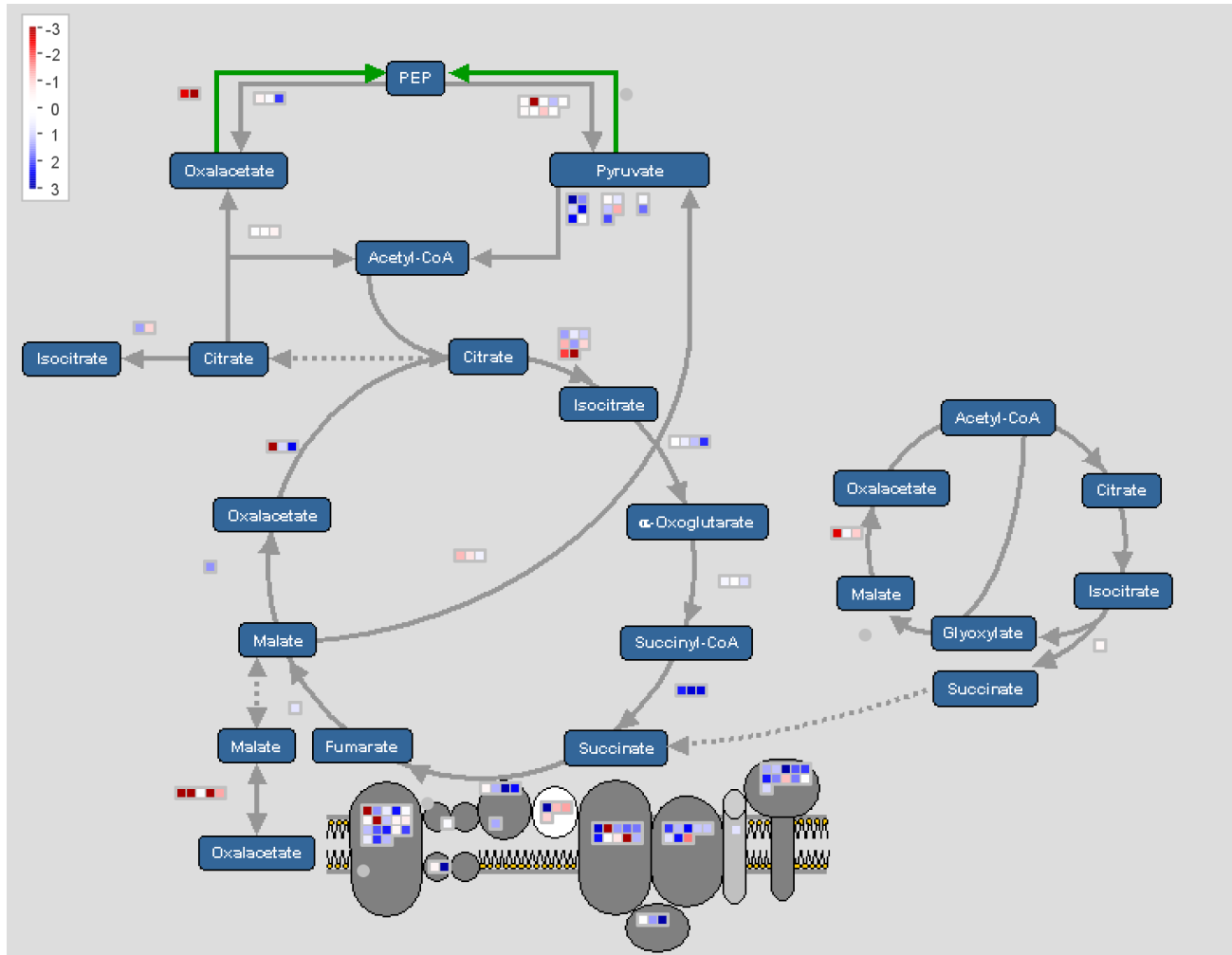

# B

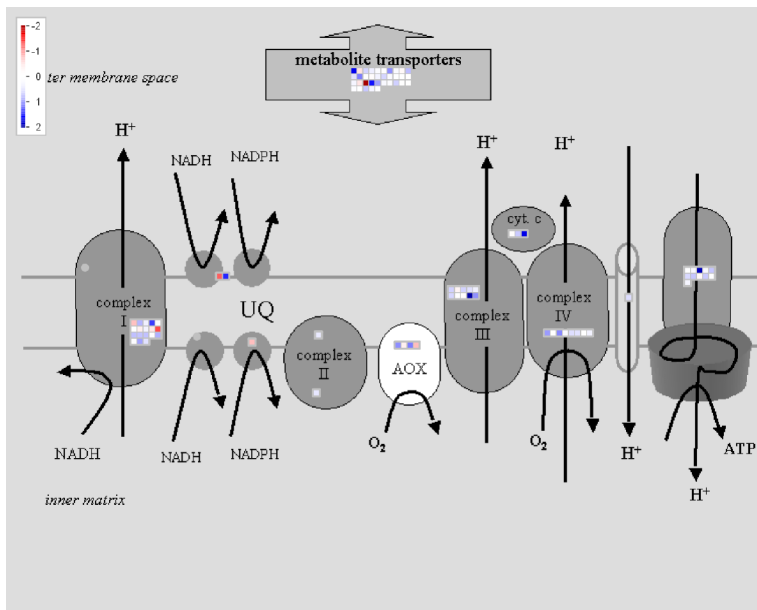

Supplement: Additional file 9: Figure S5 — Details of the TCA cycle and electron transport chain. (A) Differences in TCA cycle between meiocytes and seedlings. (B) Differences in mitochondrial electron transport chain in detail, in genes defined as meiosis genes. Scale shows log2 fold change between samples, blue = higher in meiocytes, red = lower in meiocytes. Analysis done with MapMan. [file 1471-2229-14-118-S9.pdf]
