# Supplementary material for: The transcriptome landscape of early maize meiosis
Source: BMC Plant Biol. 2014 May 3;14:118. doi: 10.1186/1471-2229-14-118 (PMC4032173; doi:10.1186/1471-2229-14-118)

Supplemental Figure 6

A

ID	description	anther RPM	meiocytes RPM	seedlings RPM
GRMZM2G109618	*Dmc1* (meiotic recombinase)	257.06	463.66	0.39
GRMZM5G839924	*Nad9* (mitochondria encoded, subunit of NADH-DH of electron transport chain)	1.41	17.16	0.12
GRMZM2G013331	ribosome-inactivating protein	0.00	0.08	0.00
GRMZM2G152958	Dihydrolipoyl dehydrogenase	0.10	0.01	0.05

B ***Dmc1***

premeiotic prelepto lepto zygo early zygo late spores

sense

anti-sense

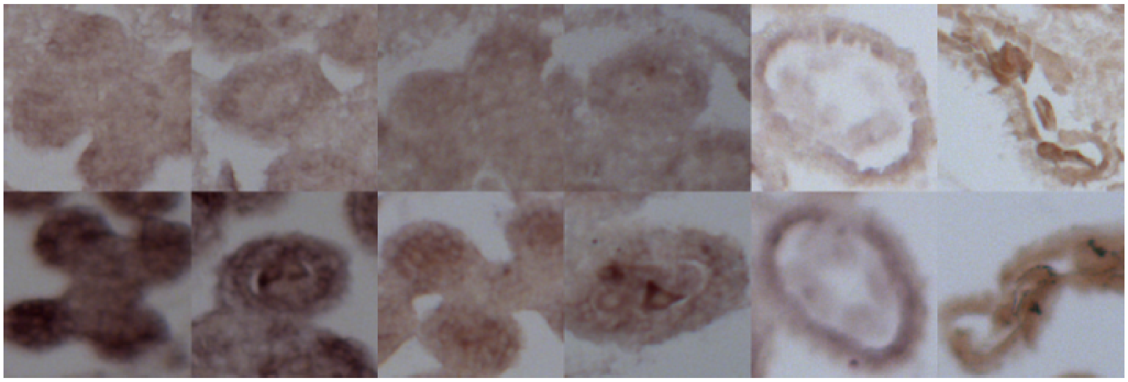

Nad (mito)

premeiotic prelepto lepto zygo early zygo late spores

sense

anti-sense

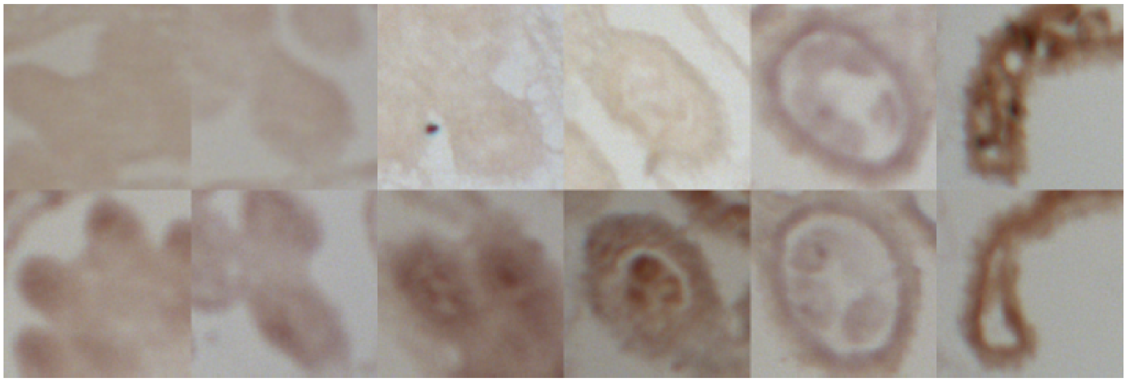

- continued on next page -

S6 B – *continued* -

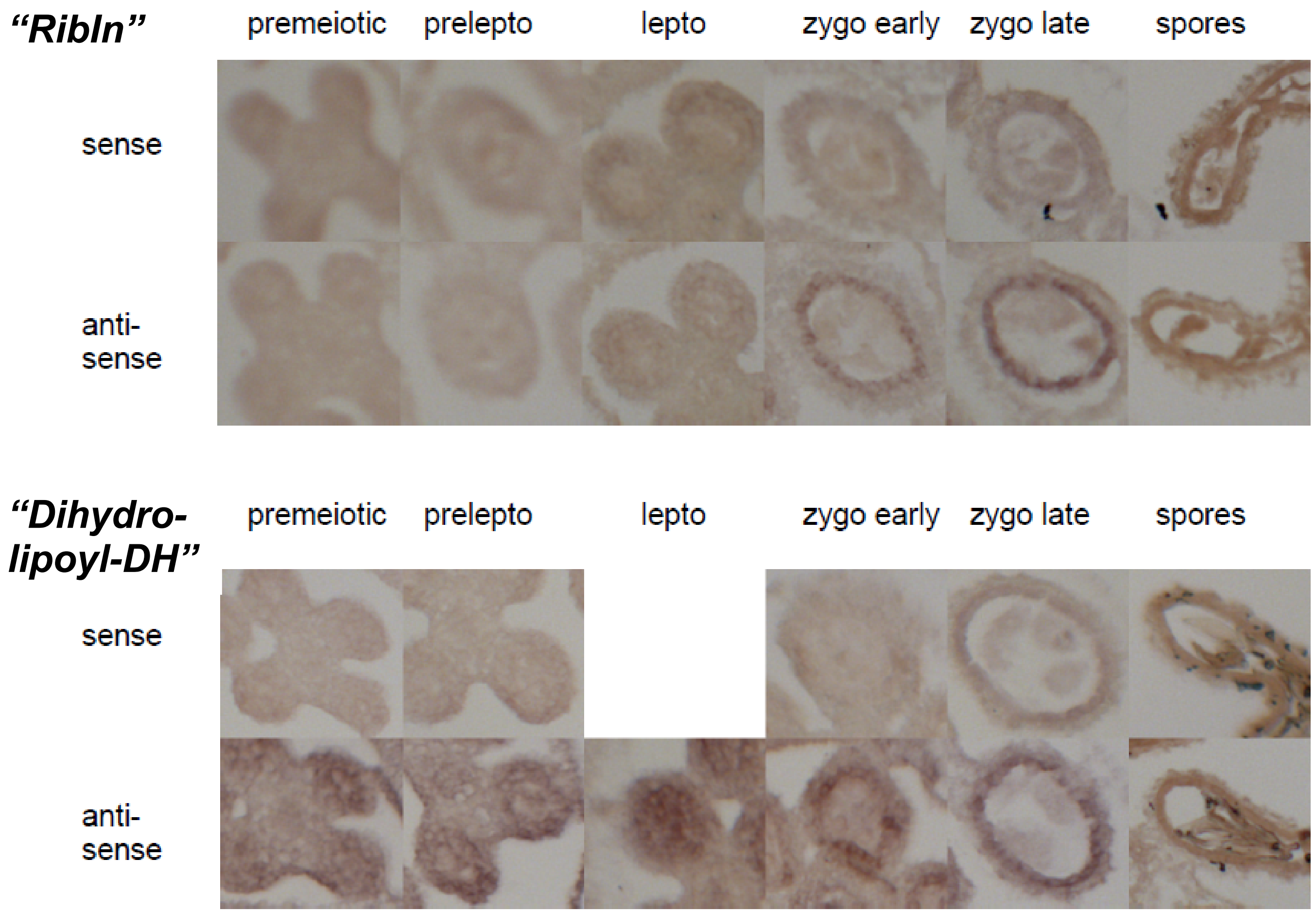

Supplement: Additional file 11: Figure S6 — RNA in situ hybridization Original images of all stages, and corresponding RNA-seq counts. (A) Table of genes used for in situ hybridization and their RPM (reads per million) counts. (B) Original in situ hybridization images. The same microscope and camera settings were used for all pictures and no editing was conducted with the cropped pictures. [file 1471-2229-14-118-S11.pdf]
